# COVID-19 in Africa: Supply chain disruptions and the role of the Africa Continental Free Trade Agreement

**DOI:** 10.7189/jogh.12.03085

**Published:** 2022-12-17

**Authors:** Jonta Kamara, Ukeme Essien

**Affiliations:** 1 King’s College London, London, UK; 2 Johns Hopkins School of Public Health, Maryland, USA

On March 11, 2020, the Director General of the World Health Organization (WHO) declared COVID-19 a pandemic [1]. There was a slow progression of COVID-19 on the African continent, which is contrary to what many had predicted at the onset of the pandemic [2-4]. The uncertainty of the pandemic led countries to impose export restrictions. Notably, European countries imposed restrictions on reagent supplies so African countries were unable to purchase them, although the funds were readily available [5]. The coronavirus pandemic has highlighted flaws in institutions and health systems across many parts of the world. Some of these flaws include structural inequities, donors’ agendas, and market forces [6].

On the African continent, the major limitations are the limited diversification in medical supply chains and lack of production capability for medical supplies leading to a high reliance on imports for health systems. It is estimated that Africa produces only 6% to 20% of its medicinal and pharmaceutical products, while the other 80% to 94% of the continent’s medical needs are met through imports [7,8]. From 2015 to 2019, intra-continental trade in Africa was only 2% compared to 47% in the Americas, 61% in Asia, 67% in Europe and 7% in Oceania, which also has a high import dependence [9]. The top five global exporters of medical supplies to Africa are responsible for providing 71% of protective equipment, 66% of disinfectants and products, and 48% of medical consumables imports to the African continent as shown in **Figure 1** [7]. The major suppliers to African countries are the European Union, China, and India [7].

**Figure 1:**
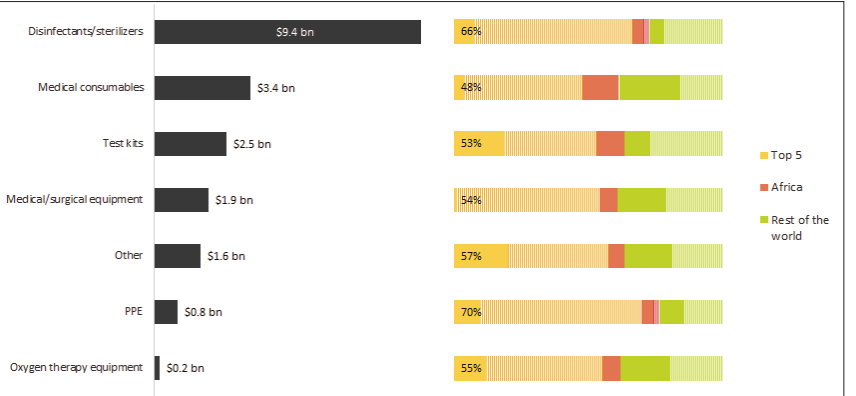
International Trade Centre (ITC) Medical Supply Imports in Africa.

In addition to countries imposing nationalistic policies during the pandemic, there was also limited global health collaboration, hence many African countries had minimal supplies to manage COVID-19. This led to the inability to scale-up testing, the inability to follow WHO testing guidelines, and shortages of protective equipment (PPE) [5]. These supply chain disruptions led to local solutions in many African countries to minimize the impact of global supply chain disruptions on their COVID-19 responses.

## CASE STUDIES

Senegal has combated the shortage in testing supplies by creating its own COVID-19 test. The Pasteur Institute in Dakar, Senegal in collaboration with a UK-based company Mologic, created an antigen-test that costs only US$1 and takes roughly 10 minutes to conduct [10]. This test is adapted to the setting in Senegal and many low- and middle-income countries as it does not require highly equipped laboratories or highly trained laboratory workers to be conducted. Although, the sensitivity of this test is diminished as meta-analysis indicates that the pooled sensitivity estimate of antigen-tests is 70% (95% CI = 69-71) while the pooled specificity estimate is 98% (95% CI = 98-98) [11]. The test uses saliva antigens and/or previous infections by blood antibodies to test. The antigen method significantly reduces the cost and time of diagnosis, as opposed to the PCR tests.

This testing platform has officially been launched in Senegal and has the capability to strengthen disease surveillance, allowing for better preparation for future epidemics. The development of this test also allows the continuation of capacity-building to occur in African countries through partnerships, which will help meet one of the Africa Centres for Disease Prevention and Control’s (Africa CDC) goals and programmes of Advancing Use of Laboratory Diagnostics [12]. This also furthers a goal of Dr Jean-Jacques Muyembe, discoverer of the Ebola virus, who sought to build research facilities in Congo [13]. This has the potential to allow for better testing and developing cures for viruses, making the process faster as samples and knowledge are processed and produced locally, therefore, strengthening epidemic preparedness and responses.

Supply chain disruptions impacted the ability to abide by WHO hand washing guidelines. To combat this, Sierra Leone developed a foot pump, while Uganda locally produced hand sanitizers. In Sierra Leone, Fomel Industries and Nationalization Industrialization Centre (FINIC) Industries, a manufacturing company, has created hands-free washing stations to follow WHO guidelines [14,15]. These devices are activated by foot pumps and play an important role in reducing the transmission of COVID-19 and other viruses. In Uganda, hand sanitizers have been made in partnership with the US Centres for Disease Prevention and Control and local health facilities, creating sanitizers in distilleries, and through the creation of local start-ups that produce sanitizers [16-18].

To combat the limited supply of PPE, in Kenya and Ghana, tailors have switched to fabric mask making [19,20]. This led to the distribution of over 50 000 masks in settlements in Nairobi, Kenya [21]. 3D masks were printed in Kenya and Malawi. Ultra Red Technologies in Kenya and iMoSyS, a tech firm in Malawi have printed 3D face masks and PPE equipment [14,22]. Ultra Red Technologies is also working to produce 3D printed reusable faces masks and 3D printed ventilator splitters [22]. These adaptive moves have reduced countries’ reliance on imported PPE and masks as domestic factories produce PPE and masks that respond to local needs. In addition to stabilizing employment rates and creating jobs, mask making creates a new economic opportunity for the textile industry.

## ROLE OF THE AFRICA CONTINENTAL FREE TRADE AGREEMENT

Although supply chain disruptions negatively impacted the availability of medical supplies, African countries were able to develop local solutions to mitigate the problem. There is the potential to scale-up these solutions by increasing intra-Africa trade of medical supplies through the launch of the Africa Continental Free Trade Agreement (AfCFTA). AfCFTA launched on January 1, 2021, and it affects 1.3 billion people in a US$3.4 trillion economic bloc of 55 countries [23]. This trade agreement allows for continent wide free trade, easing of intra-Africa trade, and is the largest free trade area after the World Trade Organization (WTO) in terms of the number of countries involved [24]. Presently, one of the known challenges for medical supply chains is the ineffective regional collaboration. For health systems to fully benefit from AfCFTA collaboration with the African Union (AU)’s 5 Regional Collaborating Centres and existing global health actors’ needs to be improved.

The COVID-19 vaccine has also displayed the high dependence of the African continent on vaccine imports. COVID-19 Vaccines Global Access (COVAX) was launched with a goal to help vaccinate individuals in low- and middle-income countries. This program alone is infective to ensure a high vaccination coverage across the continent as it would only vaccinate 20% of the African population [25]. As of April 2020, only 17% of individuals on the African continent are fully vaccinated compared to 59% of the world’s population [26]. The African continent only has 7 vaccine manufacturers that supply less than 1% of to the African continent, while the remaining 99% of vaccines come from external sources [27,28].

**Figure:**
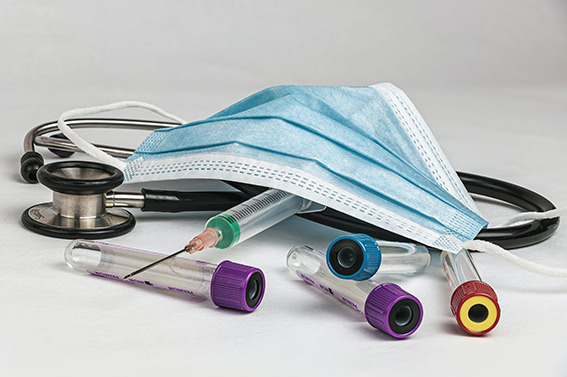
Photo: Free to use under Unsplash license. Available at: https://unsplash.com/s/photos/medical-supplies.

To minimize the unmet vaccine need, AU Member states launched the African Vaccine Acquisition Trust (AVAT), in an effort to achieve the continent’s goal of vaccinating 60% of the population [29]. On March, 28 2021, AVAT reached a historic agreement purchasing 220 million doses of the single shot Johnson & Johnson vaccine [30]. What makes this event so impactful, is that these vaccines were partly manufactured in South Africa, increasing the country’s manufacturing capabilities and hence self-reliance [30,31]. This initiative reveals that when African countries unite, they have more leverage and purchasing power in global markets. The implementation of AfCFTA and improving supply chain networks has the potential to enable the cheap and effective transportation of vaccination doses across the continent.

## CONCLUSION

The COVID-19 pandemic has made Africa’s overdependence on imports of medical supplies and global supply chains more evident. This has led countries to develop solutions to supply chain disruptions demonstrating an aspect of self-reliance in Africans health care markets. AfCFTA has a key role in increasing self-reliance and simplifying intra-Africa trade of medical supplies. This policy will strengthen continental medical supply chains and improve health systems, and help establish the new public health order for the continent.
